# Direct visualization of DNA baton pass between replication factors bound to PCNA

**DOI:** 10.1038/s41598-018-34176-2

**Published:** 2018-11-01

**Authors:** Kouta Mayanagi, Sonoko Ishino, Tsuyoshi Shirai, Takuji Oyama, Shinichi Kiyonari, Daisuke Kohda, Kosuke Morikawa, Yoshizumi Ishino

**Affiliations:** 1 Medical Institute of Bioregulation, Kyushu University, 3-1-1 Maidashi, Higashi-ku, Fukuoka, 812-8582 Japan; 2 Department of Bioscience and Biotechnology, Graduate School of Bioresource and Bioenvironmental Sciences, Kyushu University, 744 Motooka, Nishi-ku, Fukuoka, 819-0395 Japan; 3 Department of Bioscience, Nagahama Institute of Bio-Science and Technology, Tamura 1266, Nagahama, Shiga 526-0829 Japan; 4 Faculty of Life and Environmental Sciences, University of Yamanashi, 4-4-37 Takeda, Kofu, Yamanashi 400-8510 Japan; 5 Department of Gene Mechanisms, Graduate School of Biostudies, Kyoto University, Yoshida-konoemachi, Sakyo-ku, Kyoto 606-8501 Japan

## Abstract

In Eukarya and Archaea, the lagging strand synthesis is accomplished mainly by three key factors, DNA polymerase (Pol), flap endonuclease (FEN), and DNA ligase (Lig), in the DNA replication process. These three factors form important complexes with proliferating cell nuclear antigen (PCNA), thereby constructing a platform that enable each protein factor to act successively and smoothly on DNA. The structures of the Pol-PCNA-DNA and Lig-PCNA-DNA complexes alone have been visualized by single particle analysis. However, the FEN-PCNA-DNA complex structure remains unknown. In this report, we for the first time present this tertiary structure determined by single particle analysis. We also successfully visualized the structure of the FEN-Lig-PCNA-DNA complex, corresponding to a putative intermediate state between the removal of the DNA flap by FEN and the sealing of the nicked DNA by Lig. This structural study presents the direct visualization of the handing-over action, which proceeds between different replication factors on a single PCNA clamp bound to DNA. We detected a drastic conversion of the DNA from a bent form to a straight form, in addition to the dynamic motions of replication factors in the switching process.

## Introduction

Numerous proteins are involved in DNA replication, and they form a huge molecular assembly called the replisome, in which DNA clamps play important roles as a platform for these proteins. In Archaea and Eukarya, proliferating cell nuclear antigen (PCNA) is the DNA clamp, which is loaded on the primer region of DNA as a trimeric ring with the aid of the replication factor C (RFC) clamp loader. A polymerase, forming a complex with PCNA, can be constitutively tethered to the DNA strand, and thereby the enzyme successively and effectively synthesizes DNA. In addition to DNA polymerases, PCNA also interacts with various protein factors to control DNA replication, DNA repair, and cell cycle progression, and thus functions as a major conductor for the recruitment and release of these crucial players^1,2^. Over 50 proteins are known to interact with PCNA. For PCNA binding, in common, they share the conserved sequence motif called the PIP (PCNA interacting protein)-box, which is usually located in their C-terminal tails or internal flexible loops^3^.

**Figure 1:**
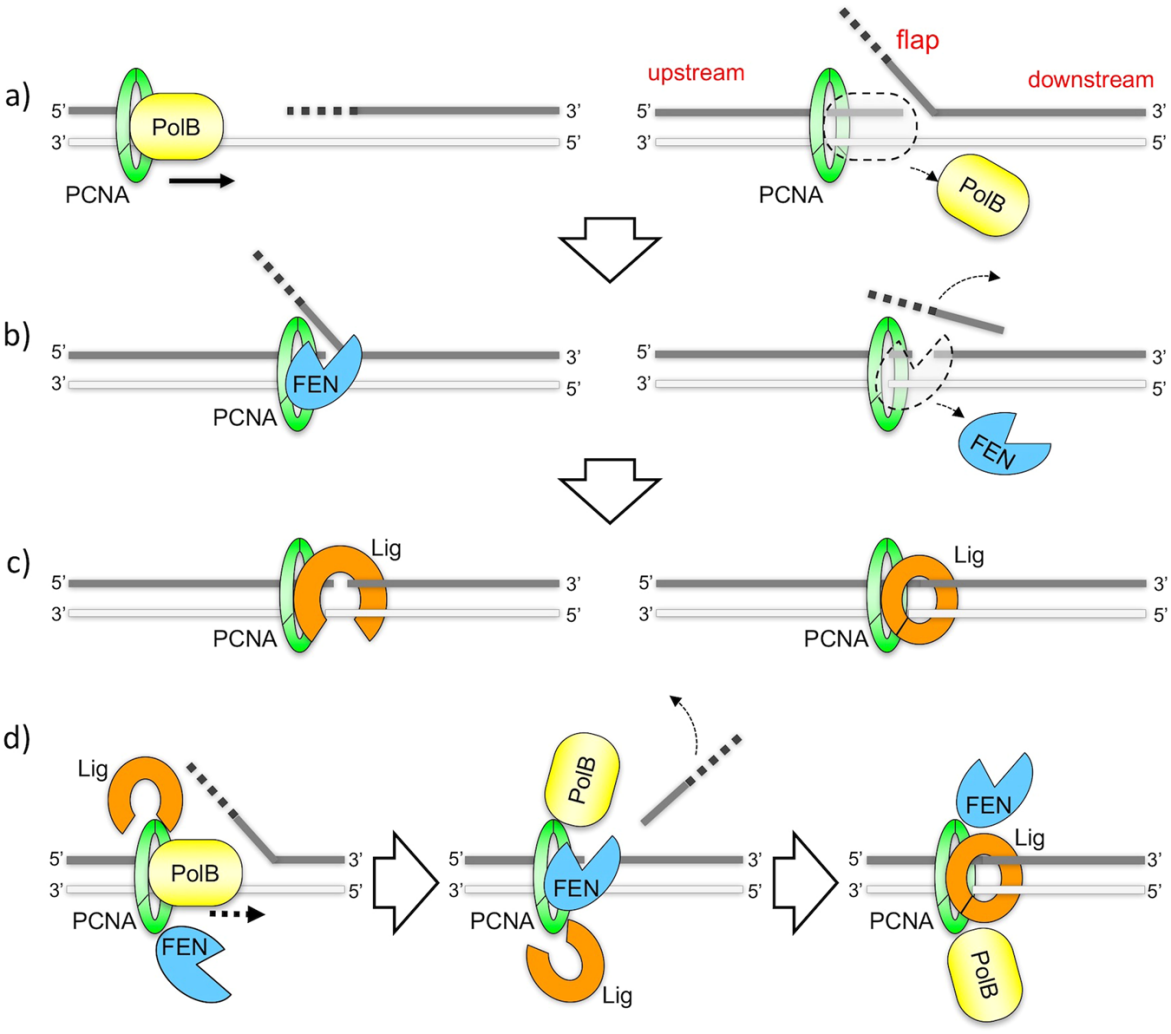
Reaction scheme of PCNA-mediated Okazaki fragment maturation (**a**–**c**) The sequential model. (**a**) Upon meeting the downstream fragment, DNA polymerase (PolB) displaces the primer region (indicated by bold dashed lines), to produce a flap DNA structure. (**b)** Next, FEN recognizes and cleaves the flap. (**c**) Finally, the nick is sealed by DNA ligase (Lig). (**d**) The standard tool belt model, in which all three of the enzymes are bound to a single PCNA trimer, and the three steps reactions are performed in order by the switching of these enzymes.

While the leading strand is replicated continuously, the lagging strand is synthesized discontinuously, as short Okazaki fragments in iterative multistep enzymatic processes (Fig. 1), and accomplished mainly by three factors, DNA polymerase (Pol), flap endonuclease (FEN), and DNA ligase (Lig). When Pol meets the downstream fragment, it displaces the primer region to produce a flap DNA structure (Fig. 1a). Next, FEN specifically recognizes and cleaves the flap (Fig. 1b), and finally the nick is sealed by Lig (Fig. 1c).

These three factors bind to PCNA through the PIP box motif. The PCNA clamp is a ring-structured molecule composed of three subunits, each with a PIP-binding region. Theoretically, three distinct protein factors could be bound to one PCNA ring. These unique structures have led to an attractive proposal termed the PCNA tool belt model^4^, which can reasonably explain the high efficiency of these sequential reactions (Fig. 1d).

Indeed, the crystal structure of human flap endonuclease-1 (FEN1, abbreviated as FEN hereafter in this paper) revealed that one FEN molecule binds to each of the three PCNA subunits^5^. Biochemical experiments^6,7^, as well as an electron microscopic study^8^, all using the *Sulfolobus solfataricus* system, generated data supporting this switching model. In contrast, a more recent biochemical study using *Saccharomyces cerevisiae* proteins suggested the sequential release and binding of replication factors^9^.

Using the DNA ligase, DNA polymerase B, and PCNA from *Pyrococcus furiosus*, we previously reported the structures of the ternary complexes of Lig-PCNA-DNA^10^ and Pol-PCNA-DNA^11^. These structures revealed that each PolB and Lig molecule occupies two of the PCNA subunits for binding. These findings appear to be at least partially inconsistent with the standard tool belt model, which assumes that three factors are simultaneously bound to a single PCNA ring. Moreover, our EM structure of the clamp loading complex, consisting of replication factor C (RFC), PCNA, and DNA, revealed that the PCNA ring is almost completely covered by RFC, thus preventing interactions with other factors^12^. Together with the fact that more than 50 proteins interact with PCNA, these observations suggest that replacements of protein factors on PCNA should occur in a sequential manner during replication.

Intriguingly, the third PCNA subunit is free in both PolB-PCNA-DNA and Lig-PCNA-DNA. This tempted us to examine the possibility that the third PCNA subunit may harbor FEN, which is essential for Okazaki fragment maturation. We investigated a model, in which the FEN-PCNA crystal structure is superimposed on these maps, and confirmed that the FEN molecule can coexist with Lig (and also coexist with PolB) on the same PCNA ring^10^.

The FEN-PCNA-flap DNA structure remains undetermined, although the structure of FEN-DNA^13^ and FEN-PCNA^5^ are solved by X-ray crystallography. Several crystal structures, including human FEN^5^ and *P. furiosus* FEN^14^, have been solved, and comparisons with the crystal structure in complex with the flap DNA substrate^13^ revealed its incision mechanism, with extraordinary specificity and efficiency by its “Measure twice, cut once” strategy^15^.

In this report, we describe the molecular architecture of FEN-PCNA-DNA, the last unknown structure among the three ternary complexes acting in Okazaki fragment maturation. We have also determined the structure of the putative intermediate state between the flap removal and DNA ligation steps, and succeeded in visualizing the handing over of the “DNA baton” from FEN to Lig.

## Results

### Preparation of the FEN-PCNA-flap DNA complex

**Figure 2:**
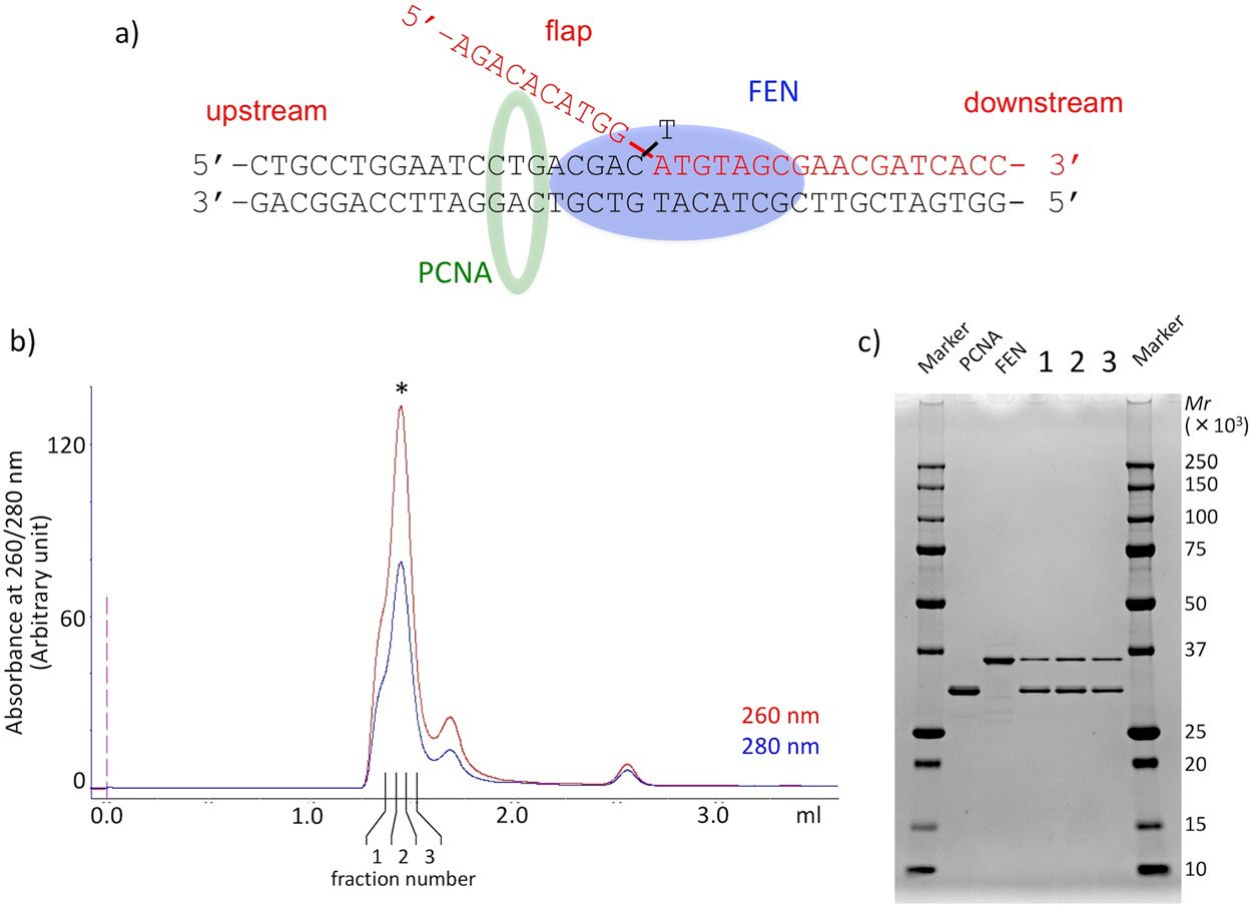
Sample preparation of FEN-PCNA-DNA (flap). (**a**) Schematic diagram of the FEN-PCNA-DNA complex with the DNA sequences used for the reconstitution. (**b**) Gel filtration chromatography of the reconstituted FEN-PCNA-DNA complex. The absorbances at 260 and 280 nm are indicated by red and blue lines, respectively. (**c**) The peak fractions indicated by the asterisk (*) in (**b**) were analyzed by gradient (10–20%) SDS-PAGE (lane 1–3). The lanes for the purified proteins used for complex reconstitution and the markers are indicated by the notations “PCNA”, “FEN”, and “Marker”, respectively.

Since FEN has optimal cleavage activity and specificity on the double flap DNA structure^16^, complex reconstitution was performed using a double flap DNA, as shown in Fig. 2a (see also Fig. S1, Substrate-A). To obtain a stable FEN-PCNA-flap DNA complex, we introduced the D175A mutation into *P. furiosus* FEN (corresponding to D181A in human FEN), which was predicted to prevent the cleavage of the 5′ flap of the DNA substrate. As expected, this mutation caused drastic suppression of the FEN endonuclease activity; however, its DNA binding ability was maintained (Fig. S2). The FEN-PCNA-flap DNA complex was then isolated by gel filtration chromatography (Fig. 2b). The main peak contained FEN and PCNA (Fig. 2c), and the absorbance ratios at 260 and 280 nm (A_260_/_280_) of the peak fraction indicated that this fraction also contains the flap DNA substrate. This highlights the suitability of our purification procedure for single particle analysis, because it avoids artificial aggregate produced by crosslinking fixation.

### Structure of the FEN-PCNA-DNA complex

**Figure 3:**
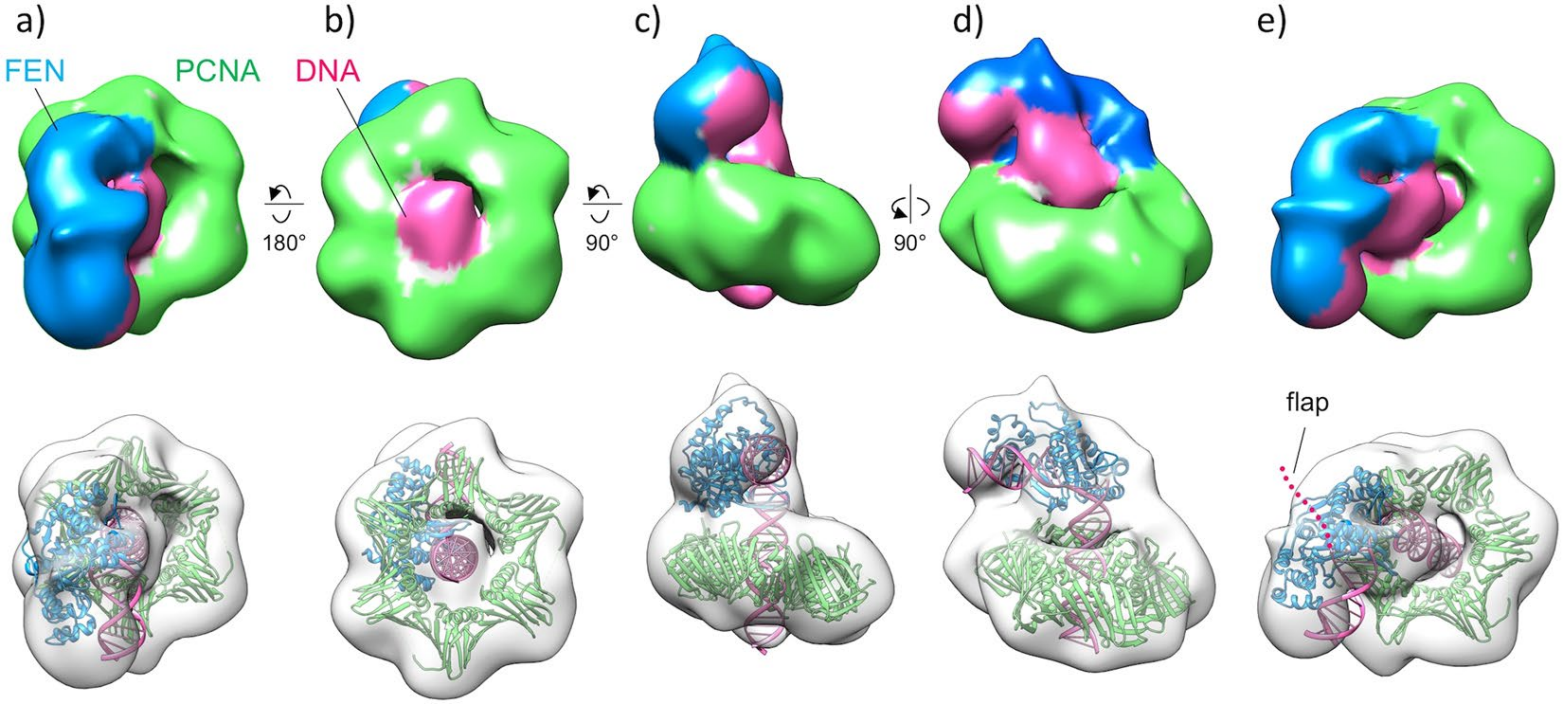
3D structure of the *P. furiosus* FEN-PCNA-DNA complex. (**a**) Top view. (**b**) Bottom view. (**c**) Front view. (**d**) Side view. (**e**) Oblique view. The EM map is colored according to the subunits. The fitted *P. furiosus* FEN and *P. furiosus* PCNA crystal structures and the atomic model of DNA are shown in sky-blue, green, and pink ribbons, respectively, in the transparent surface representation.

A representative electron microscopic image from the peak fraction is shown in Fig. S3. The particles were well dispersed, and their diameters were estimated to be about 10 nm, in good agreement with the putative size of the complex structure. The reconstructed 3D map exhibited the hexagonal ring structure derived from the PCNA trimeric ring (Fig. 3, see also Fig. S4). The resolution of the map estimated from the Fourier shell correlation (FSC) is 19 Å (Fig. S5). Notably, one side of the ring contacts a bulky region, which could be reasonably assigned to the FEN molecule. We also successfully visualized the rod-shaped density corresponding to the upstream dsDNA, which runs through the PCNA channel and extends to the FEN molecule. In contrast, we could not visualize the flap region, which is a flexible single strand of DNA that tended to be blurred out during the averaging procedure (Fig. 3e). The crystal structure of the human FEN-PCNA complex showed that a FEN molecule is bound to each of the three PCNA subunits, in quite different orientations relative to each other^5^. We examined which molecular position of FEN in the FEN-PCNA crystal (PDB ID: 1UL1) is most similar to that in our map, by comparisons with three different FEN configurations (denoted as X, Y, and Z in their paper^5^). The X configuration did not fit well with our map, but the Y and Z molecules exhibited similar positions, as shown in Fig. S6. In particular, the Y molecule was found to be almost superimposable on the bulky region in our EM map (Fig. S6b).

We next tried to fit the human FEN-DNA crystal structure^13^ (PDB ID: 3Q8M) into our map. The FEN-DNA crystal structure contained the minimum length of DNA that fully covers the FEN molecule (Fig. S7a, blue ribbon). Therefore, we created the upstream DNA model as an ideal B-form DNA (Fig. S7a, cyan ribbon) and superimposed it onto the rod density in the PCNA channel.

As shown in Fig. S7b, the human FEN-DNA crystal structure, together with the extended DNA, was consistently fitted as a rigid body into our map. However, the significant discrepancy in the orientation between the docked FEN molecule and the Y molecule in the FEN-PCNA crystal implied that FEN rotates by about twenty degrees around its major axis upon DNA binding (Fig. S7c,d).

The FEN molecule, lying along the top surface of the PCNA subunit, is quite distant from the center of the PCNA channel. Accordingly, the previous report^5^ assumed that a dynamic swinging motion of the FEN molecule towards the channel center would cause the actual interaction between FEN and DNA. However, the FEN molecule close to the Y position could interact with DNA, upon certain molecular rearrangements, such as the generation of a rectangular bend of the DNA at the branch point, the rotation of the oval-shaped FEN around its long axis and the DNA tilt in the PCNA channel (Figs 3 and S7).

Thus, the final atomic model was obtained by replacing the human FEN crystal structure with the *P. furiosus* FEN crystal structure^14^ (PDB ID: 1B43). It should be noted that the *P. furiosus* FEN lacks the helix turn helix (HTH), although the corresponding moiety of human FEN appears to substantially protrude from the map (Fig. S7b,c). Consequently, the well-fitted atomic model (Fig. 3) was constructed as explained in the Material and Methods.

### Preparation of the FEN-Lig-PCNA-nicked DNA complex

We also attempted the structural study of the FEN-Lig-PCNA-nicked DNA complex. This structure should correspond to the intermediate state between the flap cleavage and ligation reactions. Our previous analysis of the Lig-PCNA-DNA complex showed that two of the PCNA subunits are occupied by Lig. However, the third PCNA subunit was vacant, and our model building study^10^ confirmed that a single FEN molecule can indeed bind there.

**Figure 4:**
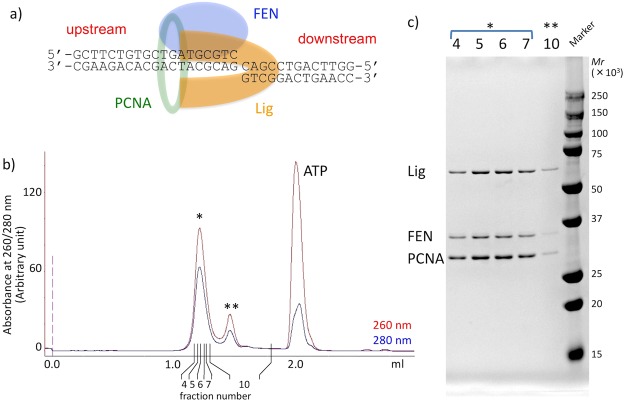
Sample preparation of FEN-Lig-PCNA-DNA (nick). (**a**) Schematic diagram of the FEN-Lig-PCNA-DNA complex with the DNA sequences used for the reconstitution. (**b**) Gel filtration chromatography of the reconstituted FEN-Lig-PCNA-DNA complex. (**c**) The peak fractions indicated by the asterisks (* and **) in (**a**) were analyzed by gradient (10–20%) SDS-PAGE (lane 4–7 and 10). Proteins corresponding to the bands are labeled on the left. Molecular weight standards are shown on the right.

We reconstituted the complex (Fig. 4a) by mixing the three purified proteins, wild type FEN, Lig, and PCNA, with a nonligatable nicked DNA (Fig. S1, Substrate-B), which contains a dideoxyribose at the 3′ terminus of the ligation site and mimics the product after flap cleavage. The gel filtration profile and SDS-PAGE analysis are shown in Fig. 4b,c, respectively. We successfully eluted the complex as a single peak, and confirmed that it contains all of the components. A representative electron microscopic image of the FEN-Lig-PCNA-DNA complex is shown in Fig. S8.

The activities of the wild type proteins used for structure analysis were confirmed by biochemical assays (Fig. S9). The flap cleavage and ligation were processed only in the presence of both FEN and Lig, and the product was increased in the presence of PCNA under the reaction conditions (Fig. S9, lanes 5 and 9).

### Structure of the FEN-Lig-PCNA-nicked DNA complex

**Figure 5:**
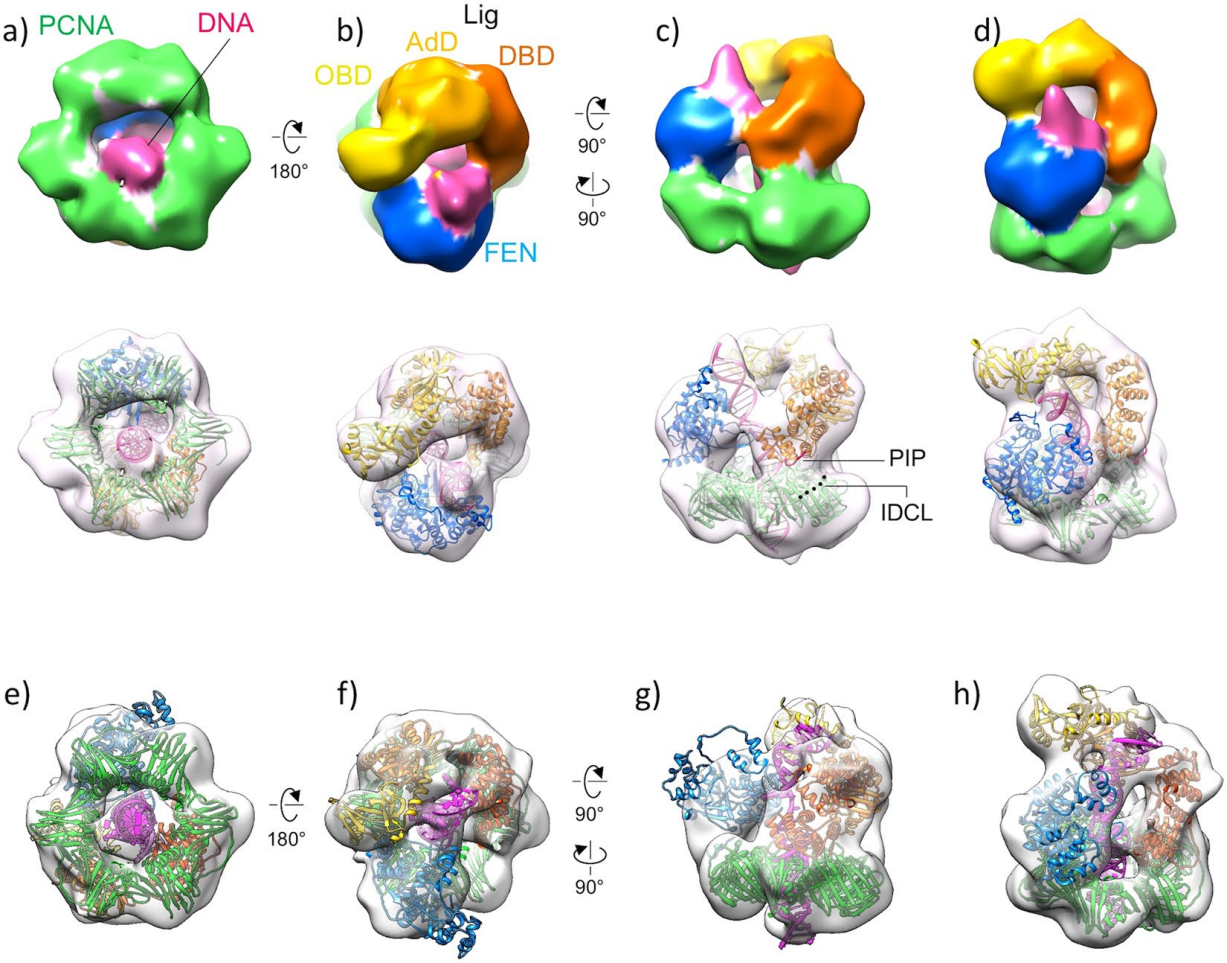
3D structure of the *P. furiosus* FEN-Lig-PCNA-DNA complex. (**a**) Bottom view, (**b**) top view, (**c**) side view, and (**d**) oblique view of the class 1 complex. The map is colored according to the components and domains. The fitted *P. furiosus* FEN and *P. furiosus* PCNA crystal structures and the atomic model of DNA are shown in sky-blue, green, and pink ribbons, respectively. The crystal structures of the *P. furiosus* Lig domains, DBD (orange), AdD (light orange), and OBD (yellow), were docked independently into the map. In (**c**), the PIP-box motif (^103^QKSFF^107^) is highlighted in red and the IDCL of PCNA is indicated with a dashed line. (**e**) Bottom view, (**f**) top view, (**g**) side view, and (**h**) oblique view of the class 2 complex. The ribbon models are colored as (**a**–**d**).

After attempting a 3D classification analysis with Relion using several class numbers, we determined that the complex could be classified mainly into two classes of 3D structures (Fig. S10). The 3D maps of the two structures, class 1 and class 2 of the FEN-Lig-PCNA-nicked DNA complexes, which were similar to each other, are shown in Fig. 5 (see also Figs S11 and S12). The resolutions of the class 1 and class 2 maps are 16 Å and 20 Å, respectively (Fig. S13). Both maps showed that the overall complex is composed of two layers: the lower corresponds to the PCNA hexagonal ring, and the upper to the two replication factors, FEN and Lig. Whereas the crystal structure of PCNA could be docked into the lower layer in a quite straightforward manner, none of the entire Lig crystal structures^17,18^ could be fitted into the upper layer as a rigid body. However, when Lig was separated into individual domains, such as the N-terminal DNA binding domain: DBD, the Adenylation domain: AdD, and the OB fold domain: OBD, each of the three domains could be docked nicely into the upper layer of the map, as in the case of Lig-PCNA-DNA^10^.

The DBD and Ad domains of Lig were bound to each of two adjacent PCNA subunits. The -QKSFF- motif, which is important for PCNA binding by *P. furiosus* Lig^19^, was located close to the C-terminus and the inter-domain connecting loop (IDCL) of PCNA (Fig. 5), indicating the high reliability of the docking model. No connections were observed between the PCNA rings and the OB fold domains in both classes. In this context, the fittings of the OB fold domain were more ambiguous than those of the N-terminal and Ad domains, suggesting the poor quality of the maps in this area. The OB fold domain of Lig is considered to be substantially mobile, as suggested from the different conformations of Lig obtained by crystallography^17,18^ and single particle analysis^10^. This higher mobility of the OB fold domain may be associated with the poor map quality of the corresponding regions.

The DNA strand in our previous Lig-PCNA-DNA structure was surrounded halfway by Lig^10^. Since the third PCNA subunit was kept free, the other side of the DNA was accessible to other factors. In contrast, both of the present FEN-Lig-PCNA-DNA structures exhibited an extra density, corresponding to the FEN molecule, and thus the DNA was completely surrounded by FEN and Lig. The main differences between the two classes were the appearance and orientation of the DNA (Fig. 5, see also Figs S11, S12 and S14). In the class 1 structure, the downstream part of the DNA protrudes upward from FEN, whereas in the class 2 structure, it extends obliquely to form a contact with Lig. The upstream part of the DNA is clearly visualized as a continuous rod in class 1, while it is hardly visible in the middle of the PCNA channel in class 2 (Fig. S14). The weak DNA density in the class 2 structure can be attributed to the negative staining method, which is not always suitable for the visualization of DNA.

At the back of PCNA, the DNA density shows a discontinuity, in the vicinity of FEN, corresponding to the position between the upstream and downstream DNA (Fig. S14). This is quite reasonable, because the DNA used in this study has a nick in the middle. The atomic models of the dsDNA containing a nick between 13 bp downstream and 19 bp upstream were well fitted into the EM map. When considering the two unpaired bases at the ds-ss junctions in the FEN-DNA crystal^13^, it is likely that the discontinuous point corresponds to the junction.

The density region, corresponding to FEN, was also different between class 1 and class 2. We investigated the relative position of FEN by docking our FEN-PCNA-DNA atomic model (Fig. 3) into both maps, by superimposing the PCNA model to the hexagonal regions. While FEN in the class 1 structure had almost the same position and orientation, it had a different orientation in the class 2 structure (Fig. S15). Based on these results, atomic models corresponding to the class 1 and class 2 structures were constructed (Fig. 5), using the same process as for FEN-PCNA-DNA. The model fittings into the two maps suggested that the orientation of FEN is more ambiguous in the class 2 model than in the class 1 model. We applied tilt validation^20^ to this class 2 map, using 10° tilted-untilted image pairs of this complex. Consequently, we obtained a cluster of plots around the expected α  = 10° nominal tilt angle, securing the validation (Fig. S16).

## Discussion

Our single particle analyses revealed the two structures of the FEN-PCNA-DNA complex and the FEN-Lig-PCNA-DNA complex, which provide important insights into the molecular mechanism of the lagging strand maturation. Although FEN and Lig are key enzymes for the maturation of Okazaki fragments, their functional states bound to both of PCNA and DNA have not been visualized yet. A similar approach was applied for the Okazaki fragment-processing holoenzyme from the archaeon *S. solfataricus*^8^. However, this crenarchaeon has a heterotrimeric PCNA clamp composed of PCNA1, 2, and 3, each of which has binding specificity to FEN, Pol, and Lig, respectively^21^. The reported models, based on maps with invisible DNA, appear to be somewhat inconsistent with our structures. The PCNA from *P. furiosus* used in this study forms a homotrimeic ring as conserved in eukaryotes, and therefore, the molecular mechanisms may be different between the two systems. Furthermore, chemical cross linking and subsequent density gradient centrifugation (GraFix method)^22^ were applied for the sample preparation from *S. solfataricus* proteins, in contrast to our samples without crosslinking. Conceivably, these differences may explain some discrepancy between the two structural studies.

The present structure of the FEN-PCNA-DNA complex is closely related to the analysis of the preceding partial structures, such as the crystal structures of the FEN-DNA and FEN-PCNA complexes. The FEN-DNA crystal structure revealed that the remarkable specificity and high efficiency of the flap DNA cleavage are achieved via the unique recognition of FEN, which induces a 100° bent at the junction on either side of the dsDNA^13^. This molecular mechanism, called the “Measure twice, cut once” strategy, aptly explains the specific and efficient mechanism of the flap cleavage reaction. However, while 12 bp of the downstream ds DNA was held by FEN, only four bp of the upstream DNA interacted with FEN in the crystal structure. A study, using the combined approach of single particle EM and molecular dynamics, indicated that essentially the same mechanism works in FEN complexed with the checkpoint clamp Rad9-Hus1-Rad1^23^.

The FEN-DNA crystal structure fits well into our FEN-PCNA-DNA EM map, where the upstream DNA passes through the PCNA channel without collision. In our structure, FEN and PCNA retain 15 to 16 bp of this DNA duplex, which would have a length approximately equivalent to that of the downstream DNA, thus suggesting that PCNA also contributes to the substrate recognition by DNA bending. Our EM map of FEN-PCNA-DNA also revealed that the FEN-PCNA crystal structure was much closer to the functional one than previously thought^5^. FEN in the Y conformation of the FEN-PCNA crystal could interact with the substrate DNA by its slight rotation (~20 deg) around its long axis, without the assumed large swing motion towards the center of the PCNA channel (Fig. 3 see also Fig. S7).

The crystal structure of the human FEN-PCNA complex apparently supported the “tool belt model”, because of the simultaneous binding of three replication factors on a single PCNA ring^5^. Although several biochemical data support this model, there are also many reports describing counter evidence, thus leading to the proposal of the “sequential model”^9^. Our previous analyses of Lig-PCNA-DNA^10^ and PolB-PCNA-DNA^11^ were not consistent with the standard tool belt model, because both complexes showed that two subunits of the PCNA ring are occupied by a single replication factor.

**Figure 6:**
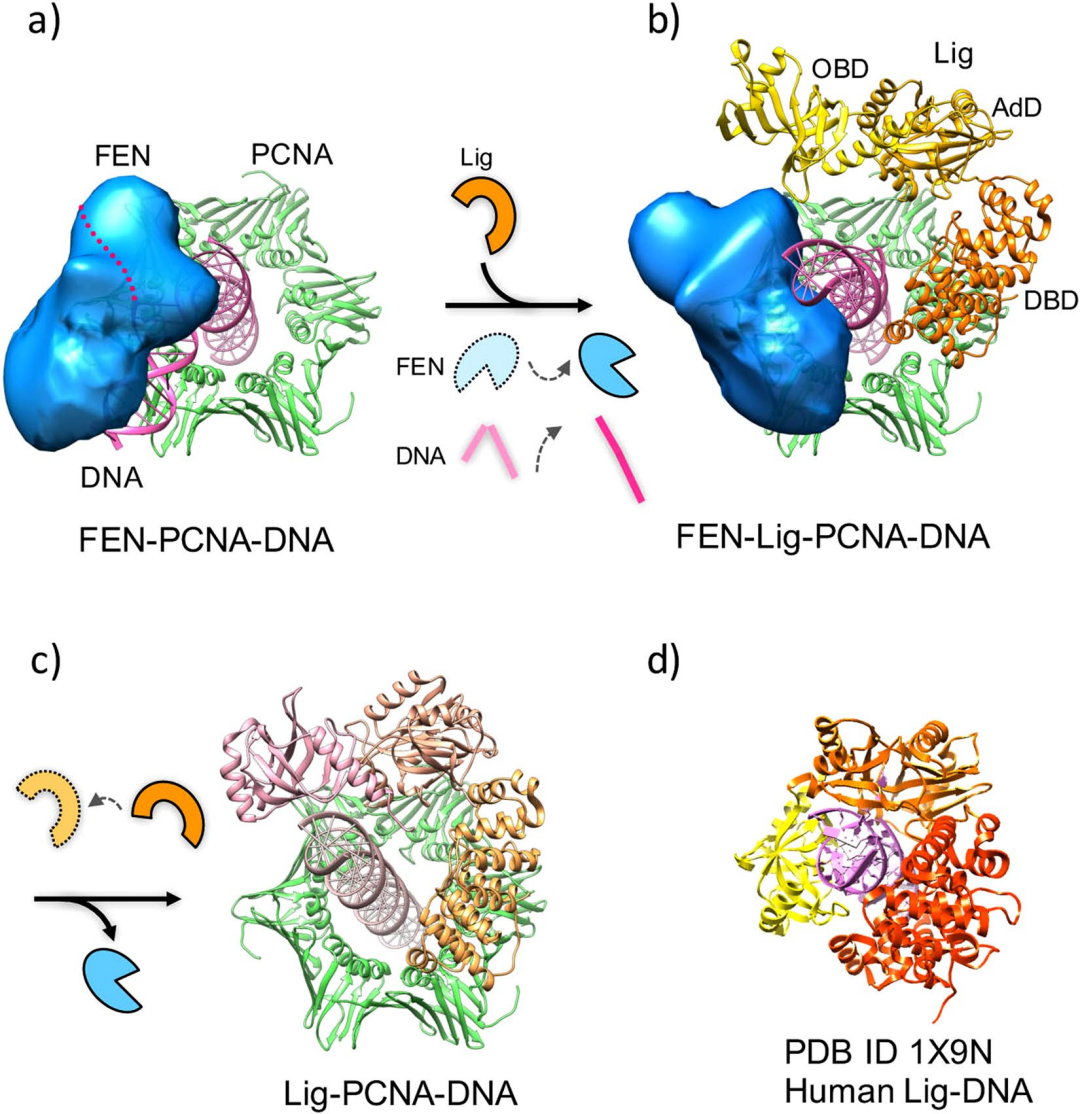
Possible mechanism of “passing DNA baton” during Okazaki fragment maturation. (**a**) The FEN-mediated flap cleavage occurs on PCNA by bending the DNA by about 100 degrees (this study). (**b**) The nicked dsDNA, generated by the action of FEN, becomes extended so that Lig contacts the DNA. FEN stays in the complex until Lig binds to the complex to form the “the handing-over” intermediate (this study). (**c**) After dissociation of FEN from the complex, Lig may form another open conformation with a small movement of the OBD towards DNA, as seen in the previous Lig-PCNA-DNA EM structure^10^. A further conformational change could occur so that Lig wraps around the DNA to form the active and closed conformation observed in the human Lig-DNA crystal structure (**d**). It is currently unclear whether PCNA remains loaded onto the DNA to support the ligation reaction by Lig.

Our EM map of the FEN-Lig-PCNA-DNA complex was not consistent with either the tool belt or sequential models^9^. Instead, the obtained structure appeared to be capturing the moment of DNA transfer from FEN to Lig; FEN remains on PCNA after its nucleolytic reaction, while it dissociates the DNA product. This step would result in the conversion of the DNA duplex from the bent to straight form (Fig. 6). The two contacts between Lig and PCNA appear to maintain the open ligase conformation, which could be regarded as an intermediate state^10^, just before DNA ligation.

Our structure, containing the single PCNA ring harboring the two factors, is consistent with the previous biochemical study^9^ in which a mutation was introduced into the PIP box binding site of PCNA, thereby preventing its binding to each of the three different replication factors, and the efficiency of Okazaki fragment maturation was measured for the mutant PCNA rings. The efficiency was not seriously affected when one PCNA subunit alone was replaced by the mutant (*i.e*. two replication factors can bind via the PIP-box motif). However, when the number of mutants in a PCNA ring increased to two (*i.e*. the number of bonds via PIP becomes one), the efficiency decreased drastically.

These results allow us to propose a model, in which the switching of FEN to Lig will occur by handing over of DNA between them, and the multiple contact sites of PCNA are utilized for the coexistence of the two factors at the moment of switching (Fig. 6). Furthermore we assume that switching between other factors would occur by similar mechanisms. The concepts of the “Hand over” model or the “Passing the DNA baton” model have previously been discussed in many reports relevant to base excision repair^24^ and replication^13^. These discussions were based on the structures of individual proteins with or without DNA. Our study provides the first direct view, where “Passing the DNA baton”^24^ is proceeding between two different replication factors.

Although the orientation of FEN could not be unambiguously determined in our structure due to the limited resolution of the map, the DNA in the FEN-Lig-PCNA-DNA complex is probably in contact with FEN in a different arrangement from that found in the human FEN-DNA crystal^13^. After the flap cleavage, the nicked DNA would have shifted (about 30 Å) from the active site of FEN, thereby converting from the 100° bent form to the straight form.

It is not possible to clarify whether FEN positively pushes the DNA to the center of channel, or Lig plays some roles in dragging the DNA. In this context, it should be noted that the complex contains the nicked DNA, which is a product mimic but not a substrate. Therefore, we cannot exclude the possibility that the cleavage of the DNA flap by the enzyme generates unknown large domain rearrangements of FEN and/or Lig. It is also possible to presume that FEN and Lig would partially contact each other during the cleavage reaction. In any event, it is likely that FEN contributes to keeping the DNA away from other factors, until Lig accesses the DNA substrate, and holds it in the straight conformation prepared for the next reaction. The Lig conformation in the FEN-Lig-PCNA-DNA complex was essentially the same as that in the Lig-PCNA-DNA complex. In order to seal the nick, Lig needs to completely wrap around DNA, and convert its structure from the open conformation, sustained by the two PCNA interaction^10^, to a closed one, as shown in the Lig-DNA crystal^17^ (Fig. 6d).

In conclusion, our study highlights the major role of PCNA to work as the universal platform, on which various replication factors closely communicate with each other to maintain the correct order of reactions in lagging strand maturation.

## Material and Methods

### Expression and purification of proteins

*P. furiosus* Lig and *P. furiosus* PCNA were purified as described previously with slight modifications^19,25^. Cloning of the genes encoding *P. furiosus* FEN and mutant proteins was performed as described in SI Material and Methods. To obtain recombinant *P. furiosus* FEN, *E. coli* BL21-CodonPlus (DE3)-RIL cells (Agilent) carrying pET-Fen were grown in LB medium containing 50 μg/ml ampicillin and 34 μg/ml chloramphenicol at 37 °C. The cells were cultured to an *A*_600_ = 0.4, and expression of the *fen* gene was induced by adding isopropyl β-D-thiogalactopyranoside (IPTG) to a final concentration of 1 mM and continuing the culture for 6 h at 37 °C. After cultivation, the cells were harvested and disrupted by sonication in buffer A (50 mM Tris-HCl (pH 8.0), 0.5 mM DTT, 0.1 mM EDTA, and 10% glycerol). The soluble cell extract was heated at 80 °C for 20 min. The heat-resistant fraction was treated with 0.15% polyethyleneimine to remove the nucleic acids. Soluble proteins were precipitated by 80% saturated ammonium sulfate. The precipitate was resuspended in buffer A containing 1.4 M (NH_4_)_2_SO_4_ and subjected to chromatography on a HiTrap Butyl HP column (GE Healthcare), which was developed with a 1.4–0 M (NH_4_)_2_SO_4_ gradient in buffer A. The fraction containing FEN was dialyzed against buffer A. The dialysate was loaded onto a HiTrap Heparin HP column (GE Healthcare), which was developed with a 0–1 M NaCl gradient in buffer A. The mutant proteins were prepared by the same procedures as the wild type (WT).

The protein concentrations were calculated by measuring the absorbance at 280 nm, with a theoretical extinction coefficient of 34,380, based on the tryptophan and tyrosine contents^26^.

### Preparation of substrate DNA

The non-labeled and the fluorescent (Cy5 or FITC)-labeled oligonucleotides were obtained from Hokkaido System Science (Sapporo, Japan) and Sigma Genosys (Tokyo, Japan). The sequences and schematic diagrams of the DNA structures are shown in Fig. S1. The double-flap DNA (Substrate-A, C, and D) was prepared by annealing synthetic oligonucleotides in 50 mM Tris-HCl (pH 8.0), and 50 mM NaCl. The annealed DNA (Substrate-A) used in the FEN-PCNA-DNA complex was further purified by gel filtration on a 2.4 ml Superdex 200 PC 3.2/30 column, with elution buffer containing 50 mM Tris-HCl (pH 8.0), and 50 mM NaCl. The nicked dsDNA (Substrate-B) was prepared by annealing synthetic oligonucleotides in 10 mM Tris-HCl (pH 8.0), and 5 mM MgCl_2_. The annealed DNA was further purified by gel filtration on a 2.4 ml Superdex 200 PC 3.2/30 column, with elution buffer containing 10 mM Tris-HCl (pH 8.0), and 5 mM MgCl_2_.

### EM sample preparation

The purified FEN, PCNA, and flap DNA (Fig. S1, Substrate-A) were mixed and incubated in reconstitution buffer, containing 50 mM Tris-HCl (pH 8.0), and 50 mM NaCl, at 37 °C for 10 min. The reconstituted FEN-PCNA-DNA complex was loaded onto a Superdex 200 5/150 (GE Healthcare) gel filtration column equilibrated with 50 mM Tris-HCl (pH 8.0), and 50 mM NaCl, and eluted with the same buffer. To purify the FEN-Lig-PCNA-DNA complex, purified proteins and nicked DNA were mixed and incubated in buffer, containing 20 mM MES (pH 6.5), 50 mM NaCl, 0.1 mM ATP, and 5 mM MgCl_2_ at 50 °C for 10 min. The mixture was loaded onto a Superdex 200 3.2/300 (GE Healthcare) gel filtration column equilibrated with elution buffer, containing 20 mM MES (pH 6.5), 50 mM NaCl, and 5 mM MgCl_2_. The protein compositions of both complexes, eluted at the peak fractions, were analyzed by SDS-PAGE.

For the negatively stained specimen, a 3 μl aliquot of sample solution was applied to a glow discharged continuous thin-carbon film supported by a copper grid, left for 1 min, and then stained with 3 drops of on-ice-cooled 2% uranyl acetate.

### Electron microscopy and single particle image analysis

Images of negatively stained specimens were examined using a T20 electron microscope (FEI) operated at an accelerating voltage of 200 kV. Images were recorded by an Eagle 2k CCD camera (FEI), with a pixel size of 2.6 Å/pixel. The magnification of the images was calibrated using tobacco mosaic virus as a reference sample. A low dose system was used to reduce the electron radiation damage of the sample.

Electron microscopic images of FEN-PCNA-DNA were examined, and complex images were picked using either the BOXER program in EMAN^27^, or the Relion viewer. Image analysis and 3D structure analysis were performed using EMAN^27^ and Relion^28,29^. A total of 44,412 images of FEN-PCNA-flap DNA were picked and analyzed by the Class2D procedure in Relion, and “bad particles”, such as protein monomers, aggregates, spurious particles, and particles with two PCNA rings, were removed. The remaining 31,570 particles were further analyzed for the 3D classification procedure. The final 3D map was calculated from 11,298 particle images by the Refine3D procedure in Relion. Post-processing procedure in Relion, which will apply solvent masking and B-factor sharpening, was not performed to the refined map.

The 3D maps of FEN-Lig-PCNA-nicked DNA (FEN-Lig-PCNA-DNA) were analyzed, essentially with the same procedure as that for the FEN-PCNA-DNA complex. The details are described in the caption of Fig. S10. The refined maps were also not treated with the Post-processing procedure in Relion. The initial refinement of class 2 structure of FEN-Lig-PCNA-DNA exhibited signs of over-refinement, thus the final map was obtained by limiting the resolution to 20 Å during the refinement process.

The visualization of the 3D map and the manual fitting of the crystal structures into the map were performed with the Chimera software^30^. The atomic models of FEN-PCNA-DNA and FEN-Lig-PCNA-DNA were constructed via compiling crystal structures, similarly to the method previously applied for the Lig-PCNA-DNA^10^ and Pol-PCNA-DNA^11^ modelings. The appropriate modeling parts/templates were sought by using the SIRD database^11^, and the PDB entries 1B43, 2CFM, and 1ISQ were selected as the models for FEN1, Ligase, and PCNA, respectively. The templates for the interfaces between FEN1-DNA, FEN1-PCNA, and Lig-PCNA were the PDB entries 3Q8M, 1UL1, and 3P87, respectively. For the FEN-PCNA-DNA model, FEN1(1B43) was placed on PCNA (biological unit of 1ISQ) by referring to 1UL1. DNA molecules were introduced by superimposing 3Q8M, and the nucleotide sequence was replaced with that used in the present study. The PIP-box of FEN1 was modeled by referring to 1UL1. The entire model was then fitted into the density map by recurrently applying manual modeling and refinement with PHENIX^31^. The FEN-Lig-PCNA-DNA model was constructed by placing Lig with reference to the previously reported Lig-PCNA-DNA model^10^, and adjusting the positions and orientations of the DBD, AdD, and OB fold domains. The structure of the PIP-box motif (^103^QKSFF^107^) of Lig was revised by referring to the similar motif of RNase H2B (^297^IDTFF^301^ in 3P87).

The tilt validation^20^ of the FEN-Lig-PCNA-DNA map was performed, using programs e2RCTboxer and e2tiltvalidate, implemented in EMAN2.1^32^. The same validation was not applied to the FEN-PCNA-DNA map, considering the small complex size (150k) and the local three-fold symmetry due to the PCNA, which would disturb this method^33^. It should be also noted that this method was originally developed for cryo-electron microscopy and is not necessarily suitable for negatively stained samples^34^.

## Electronic supplementary material

Supplementary Info

## Data Availability

The data that support the findings of this study are available from the corresponding author upon request.
