# Supplementary material for: Direct visualization of DNA baton pass between replication factors bound to PCNA
Source: Sci Rep. 2018 Nov 1;8:16209. doi: 10.1038/s41598-018-34176-2 (PMC6212441; doi:10.1038/s41598-018-34176-2)
Supplement: Supplementary file 1 — Supplementary Info [file 41598_2018_34176_MOESM1_ESM.pdf]

SUPPLEMENTARY INFORMATION

Direct visualization of DNA baton pass between replication factors bound to PCNA

Kouta Mayanagi, Sonoko Ishino, Tsuyoshi Shirai, Takuji Oyama, Shinichi Kiyonari, Daisuke Kohda, Kosuke Morikawa, & Yoshizumi Ishino

Supplemental Material & Methods

Cloning of the genes encoding *P. furiosus* FEN and its mutant proteins

The flap endonuclease (*fen*) gene in *P. furiosus* (PF_RS07095) was amplified by PCR directly from *P. furiosus* genomic DNA, using the primers fen-F (5′-GCGTCACATATGGGTGTCCCAATTGGTGAG-3′) and fen-R (5′-CGTCAGGATCCTTATCTCTTGAACCAACTT-3′). The amplified gene was cloned into the pGEM-T Easy vector (Promega), and its nucleotide sequence was confirmed. The cloned genes were excised by *Nde*I-*Bam*HI and inserted into the corresponding site of pET-21a(+) (Novagen). The resultant plasmid was designated as pET-Fen. To construct the expression plasmids for the mutant FEN D175A, PCR-mediated mutagenesis was performed with a QuikChange II Site-Directed Mutagenesis Kit (Agilent) using pET-Fen as the template. The primers used for each mutagenesis are fenD175A-F: 5′-GGCTAGTCAAGATTACGCTTCCCTACTTTTTGG-3′ and fenD175A-R: 5′-CCAAAAAGTAGGGAAGCGTAATCTTGACTAGCC-3′, (the underlined nucleotides represent the alanine-substituted site). The designed mutation was confirmed by nucleotide sequencing.

Fig. S1

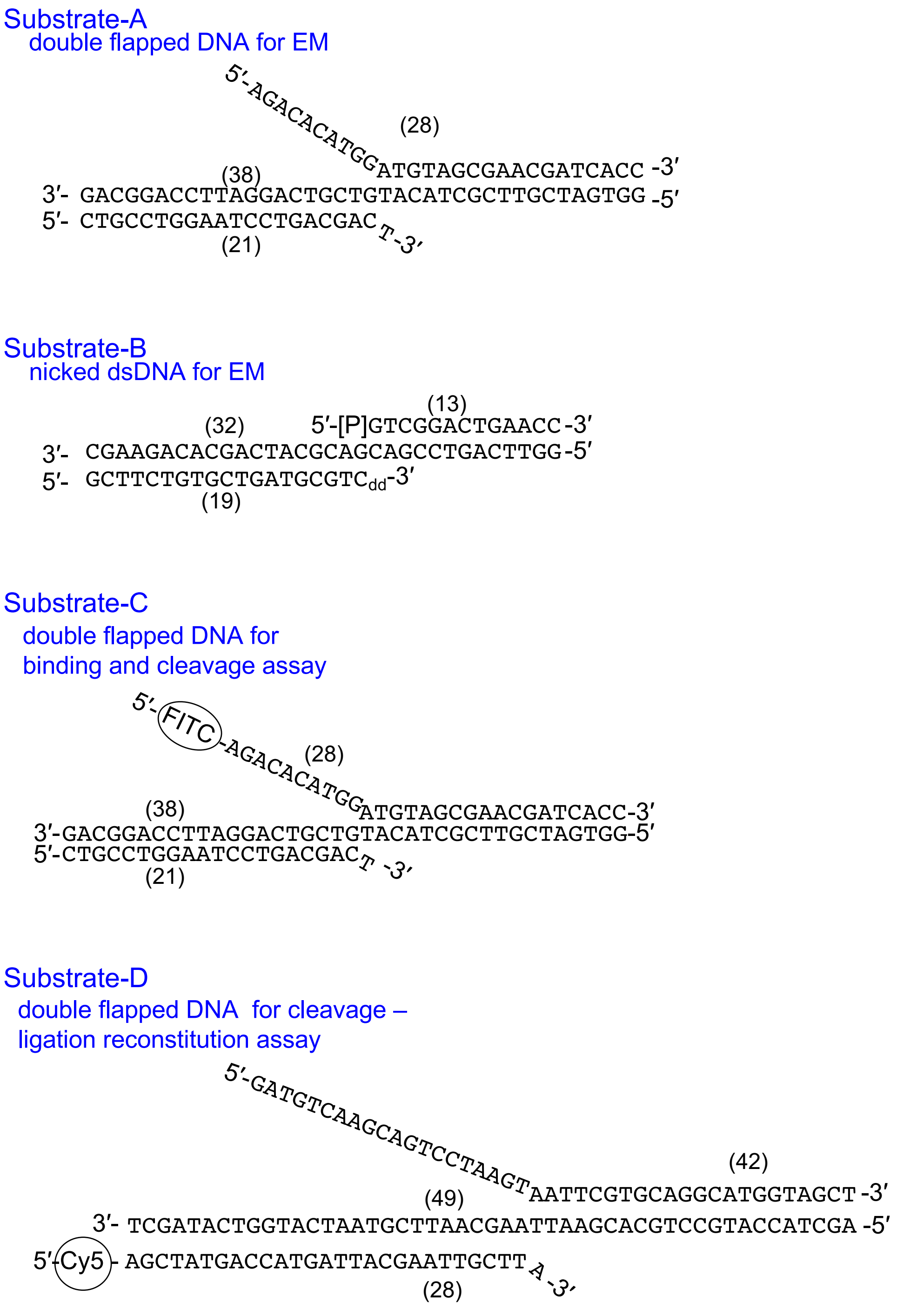

Schematic drawing of the structured DNA used in this study

The sites labeled by fluorescein are indicated by circles. The phosphate at the 5′-terminus is indicated by [P]. The dideoxyribose at the 3′-terminus is indicated by dd. The numbers in parentheses indicate the lengths of the oligonucleotides.

Fig. S2

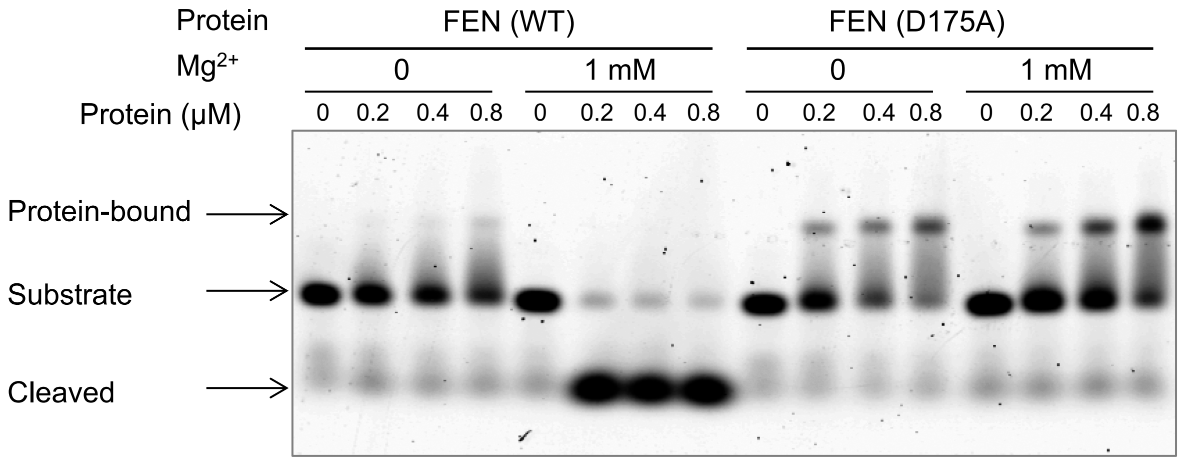

FEN activity with the double flapped DNA

The indicated amounts of the wild type (WT) and the mutant (D175A) were incubated with 5 nM Substrate-C, in 50 mM Tris-HCl, pH 7.5, 1 mM DTT, 0.01% Tween 20, and 0.1 mg/ml bovine serum albumin (BSA), at 37° for 5 min in the presence or absence of 1 mM Mg^{2+}. The reaction mixture was mixed with 2.5% Ficoll, separated by 6% native PAGE in TBE buffer, and visualized by a Typhoon Trio+ image analyzer (GE Healthcare).

Fig. S3

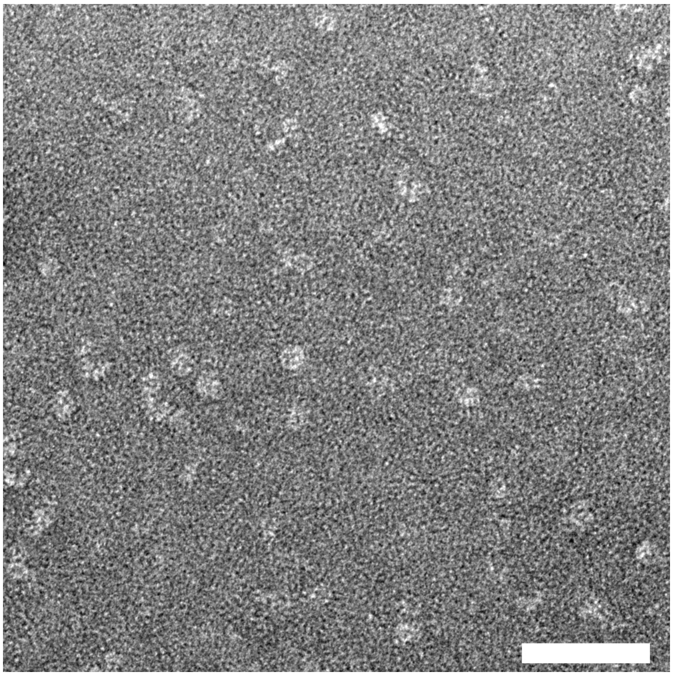

Representative electron microscopic image of the negatively stained the FEN-PCNA-DNA complex

The white scale bar represents 50 nm.

Fig. S4

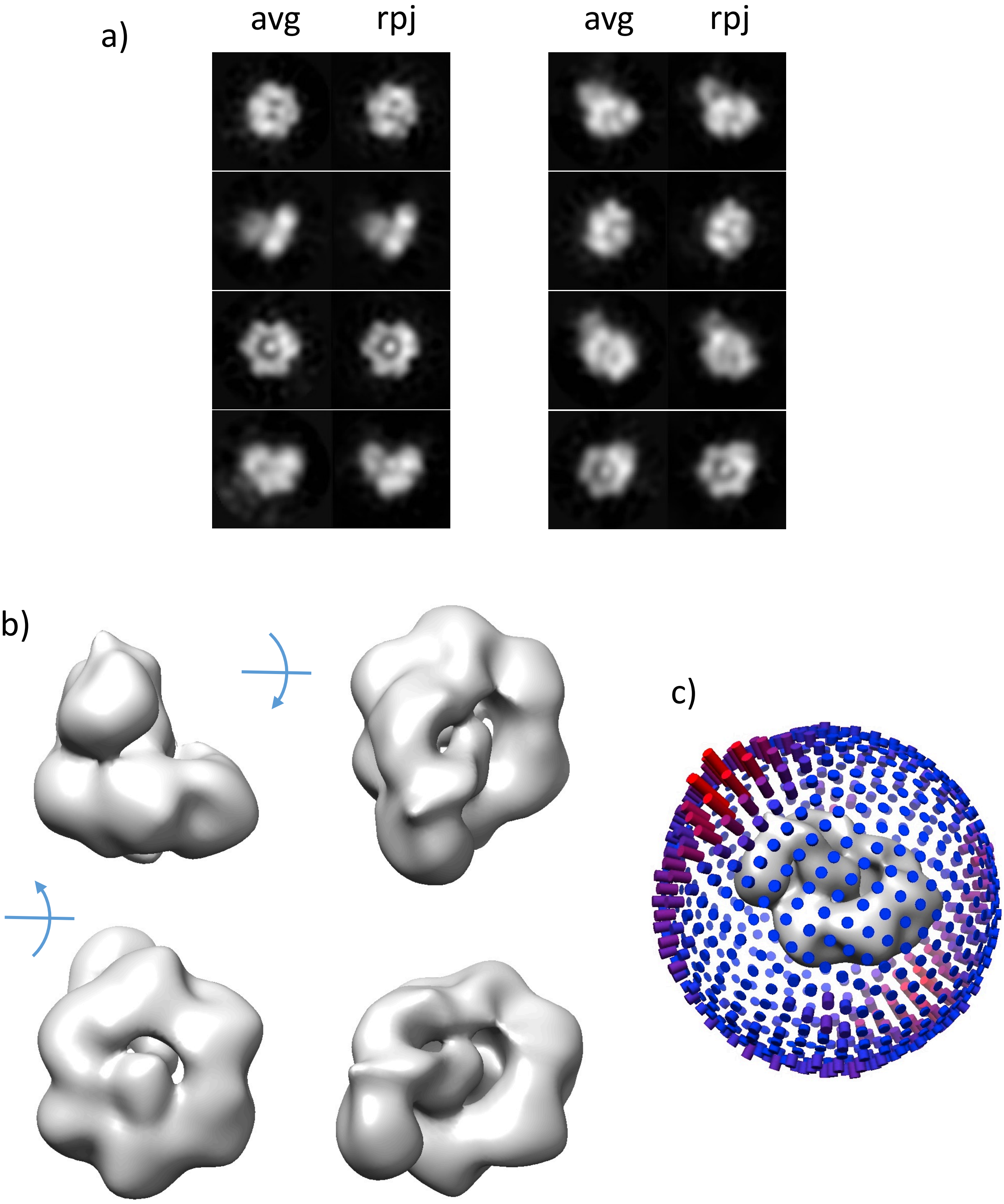

Validation of the FEN-PCNA-DNA complex structure analysis

a) Comparison between reference-free class averages (avg: left) and re-projections (rpj: right) of the refined map of FEN-PCNA-DNA. The height of each image box is 18.7 nm. b) The 3D map of the FEN-PCNA-DNA complex. . Front view (upper left), top view (upper right), bottom view (lower left), and oblique view (lower left). c) Angular distribution plot for the refined map.

Fig. S5

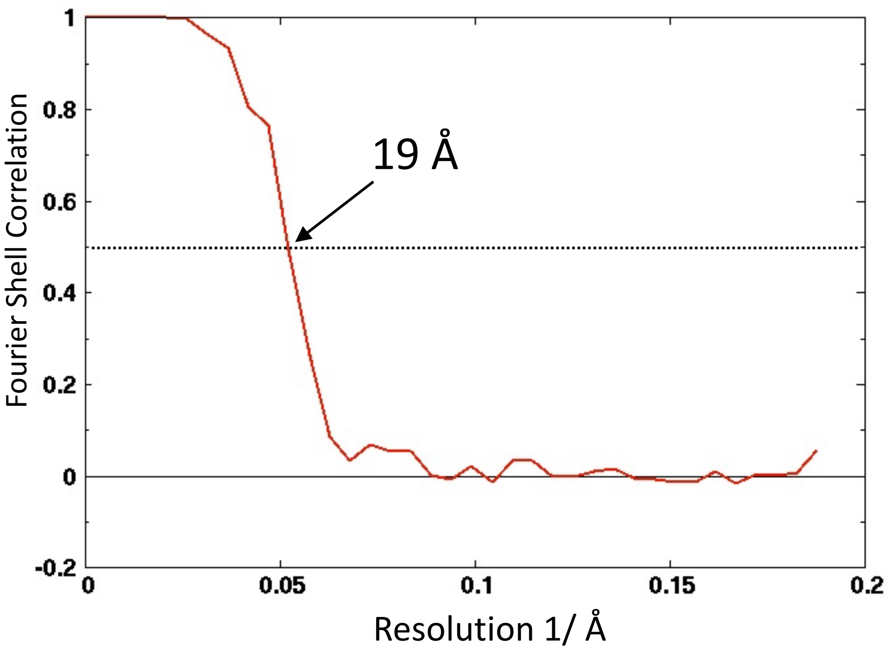

Fourier shell correlation for the FEN-PCNA-DNA complex

The correlation between two independently refined halves of the data is indicated by the solid red line, (gold-standard FSC). The resolution estimated at a correlation of 0.5, is 19 Å.

Fig. S6

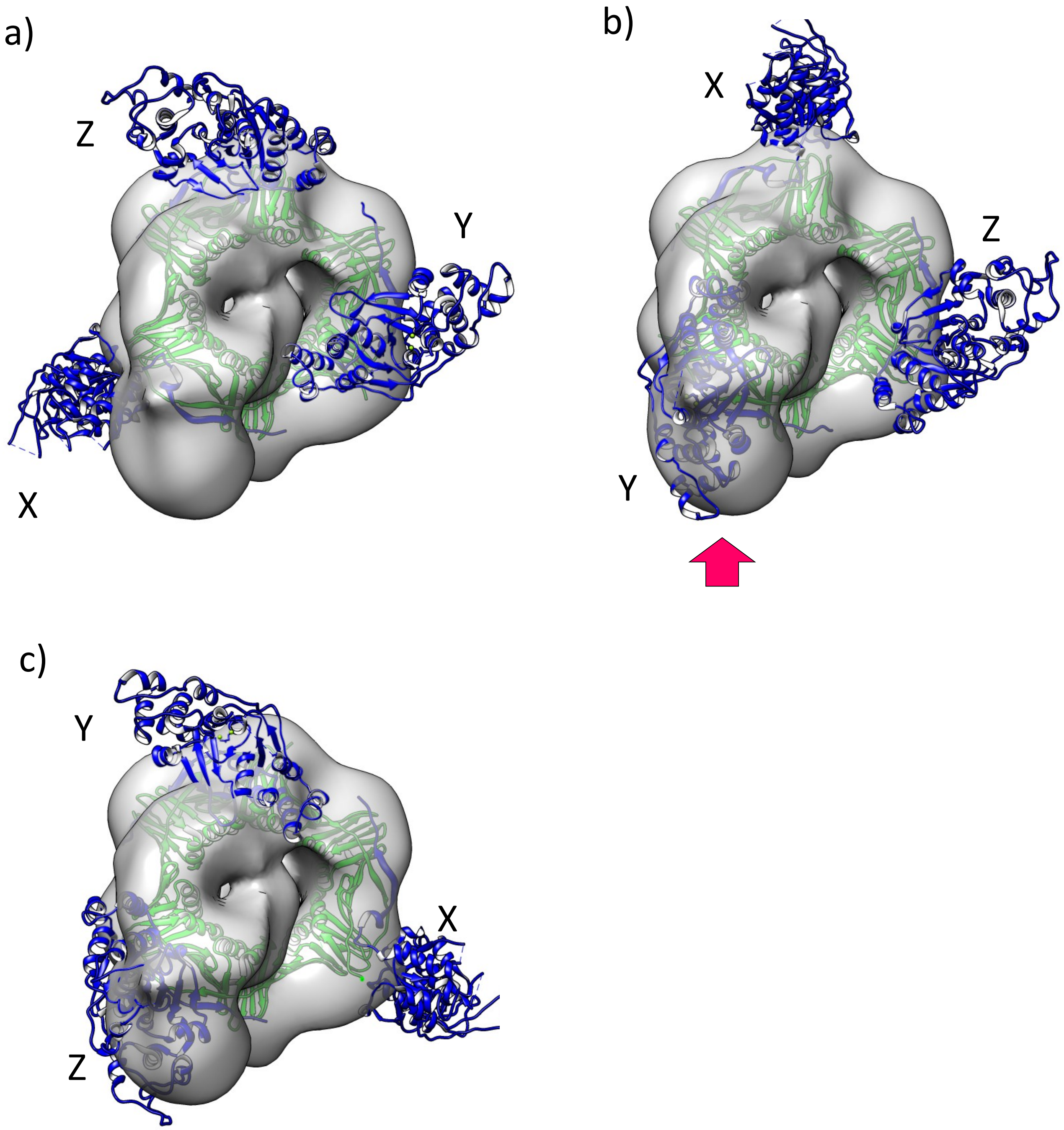

Comparison of the FEN-PCNA-DNA complex with the FEN-PCNA crystal structure

The FEN-PCNA structure was fitted to the EM map by placing PCNA into the hexagonal ring region of the map. We generated three pattern fittings and evaluated which was the best fit for FEN. PCNA and FEN molecules are shown by green and blue ribbon models, respectively. The labels X, Y, and Z indicate the three different positions of FEN, conforming with the notations of the FEN-PCNA crystal structure [5]. The Y molecule exhibited the best fit with the map, as indicated by the red arrow in b).

Fig. S7

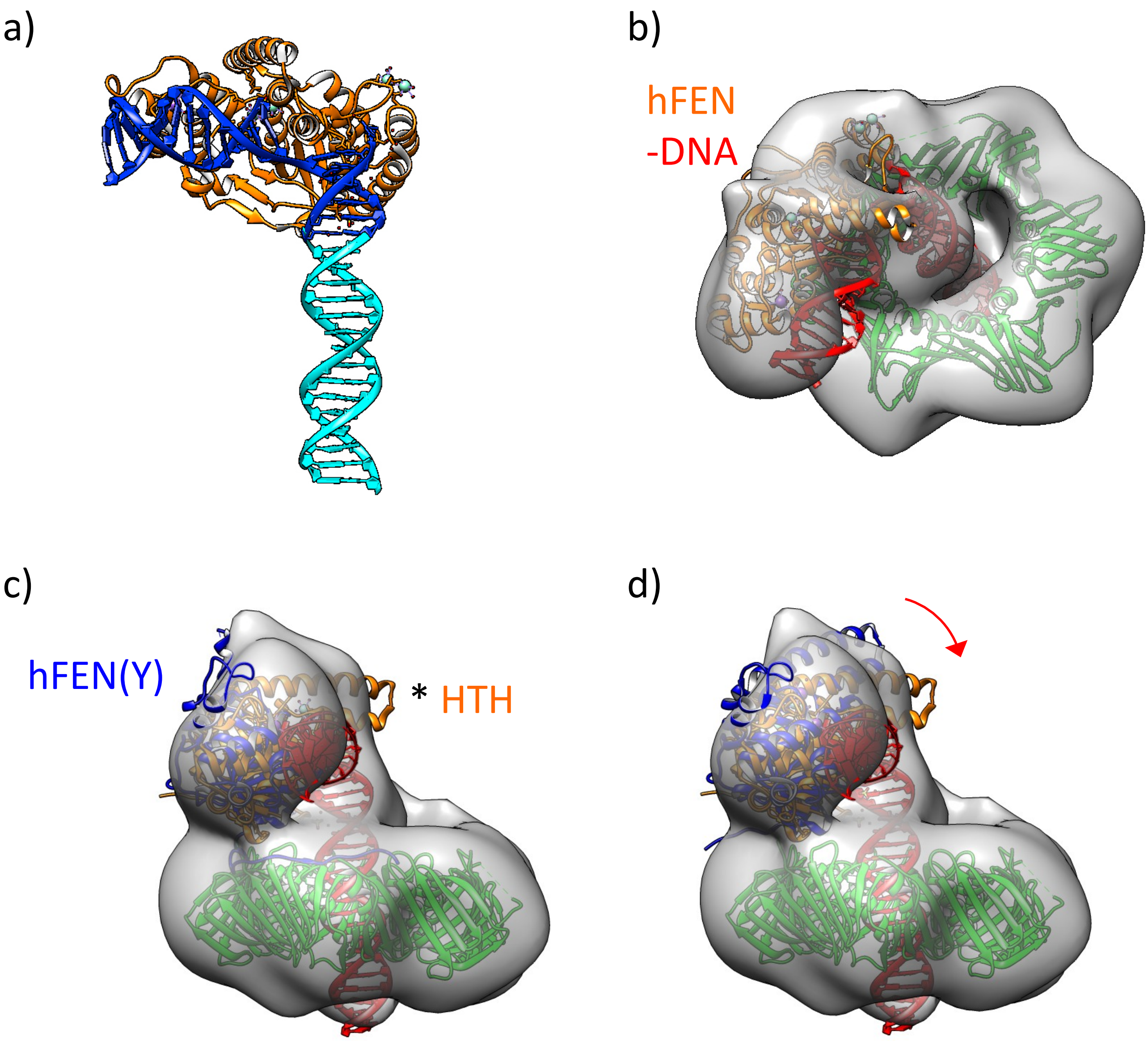

Atomic model building of the FEN-PCNA-DNA complex

a) The atomic model of the human FEN-DNA complex. human FEN (hFEN) and DNA in the crystal structure are shown in orange and blue ribbons, respectively. The upstream DNA was extended by 19 bp (cyan ribbon), for model building. b) The crystal structures of PfuPCNA (green) and hFEN-DNA shown in a) are docked into the EM map. DNA is shown as a red ribbon model. c) The hFEN in the Y position (blue ribbon) is superimposed on the hFEN-DNA-PCNA model shown in b). The helix-turn-helix (HTH indicated by *) in the hFEN-DNA complex is protruding from the map. This HTH is disordered in the FEN molecule (Y) in the hFEN-PCNA crystal. d) hFEN (Y) in c) is replaced by hFEN of the hFEN-DNA crystal structure, for clarity. A rotation of about 20° around the long axis of FEN was observed.

Fig. S8

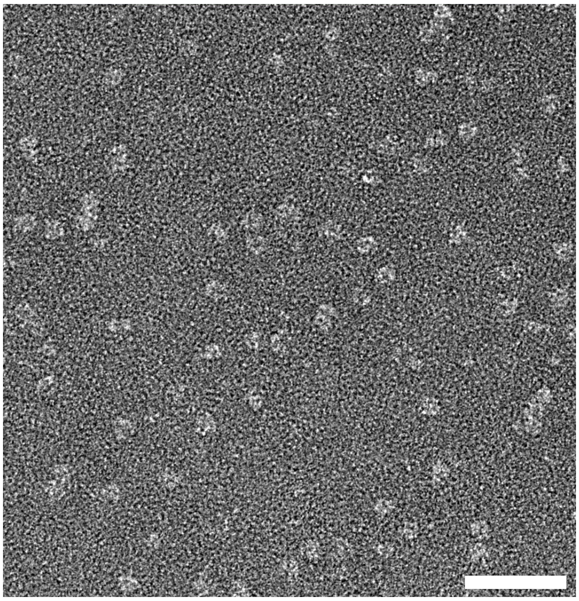

Representative electron microscopic image of the negatively stained FEN-Lig-PCNA-DNA complex

The white scale bar represents 50 nm.

Fig. S9

a)

b)

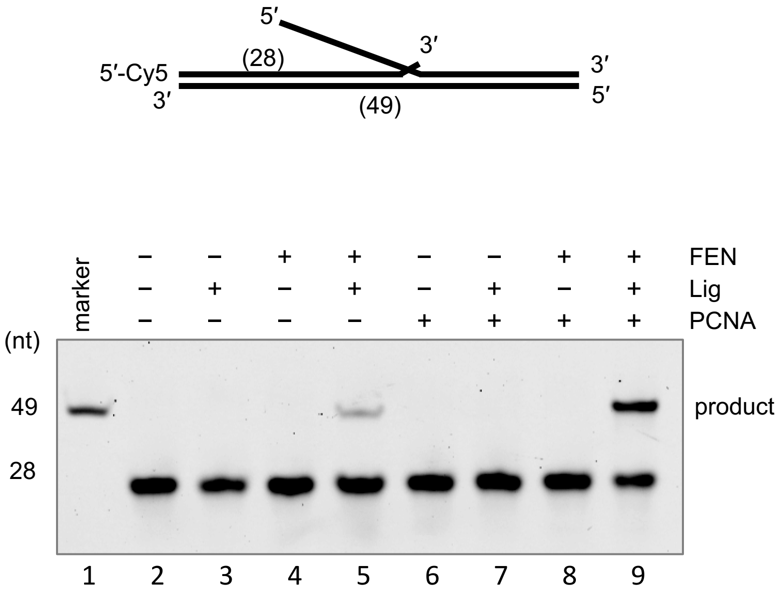

Sequential reactions catalyzed by FEN and Lig

a) Schematic drawing of the double flap substrate. Fluorescent labeling (Cy5) at the 5′ terminus of the strand is indicated. b) FEN and Lig (0.1 μM each) were incubated with 10 nM of double flap substrate (Substrate-D) in the presence or absence of 0.1 μM PCNA (as the trimer) in a reaction mixture, containing 25 mM Tris-HCl, pH 7.6, 5 mM $MgCl_2$, 0.1 mM ATP, 1 mM DTT, 0.05% Tween 20, and 0.15 M KCl, at 60 °C for 15 min. The reaction was terminated with a two-fold volume of stop solution (98% formamide, 10 mM EDTA, and 0.1% Orange G). The products were separated by 8 M urea-8% PAGE in TBE buffer and visualized with a Typhoon Trio+ image analyzer (GE Healthcare). A size marker of the product was also loaded.

Fig. S10

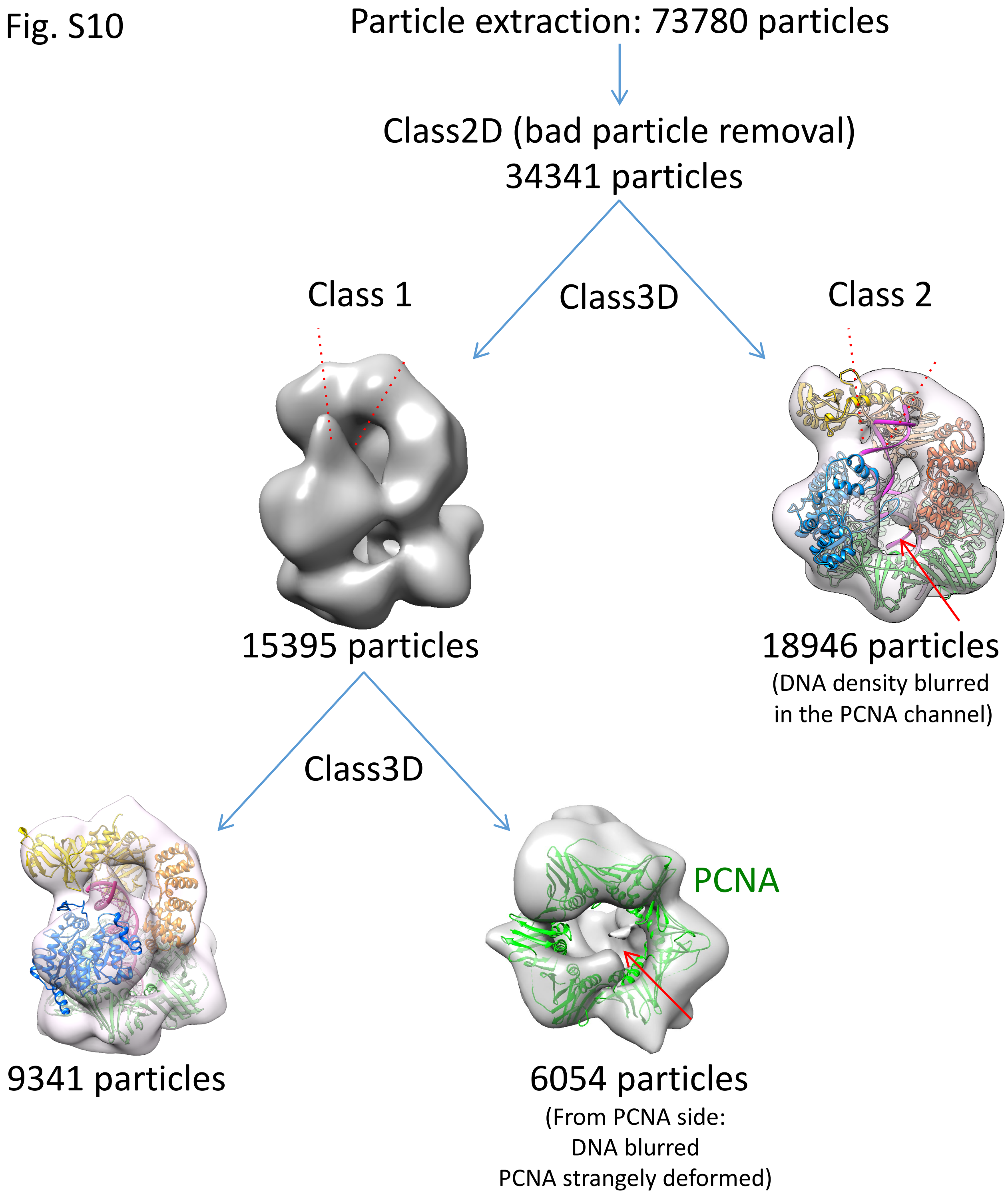

3D model reconstruction of FEN-Lig-PCNA-DNA

Using Relion 73,780 particles were picked automatically. After 2D classification, spurious particles, aggregates, and protein monomer images were removed, and the subsequent 3D classification was performed using the remaining 34,341 particles. The 3D classification analysis in Relion generated mainly two classes of the complex. Class1 and class 2 contained 15,395 and 18,946 particles, respectively. Overall, both classes showed similar shapes, but careful inspection revealed that class 2 structure exhibited a discontinuous DNA density in the PCNA channel. The class 1 complex, with a continuous DNA density in the PCNA channel, was subjected to another round of 3D classification analysis. The final 3D structure of class 1, obtained from 9,341 particles exhibited an improved rod-shaped DNA density, while 6,054 particles were discarded due to highly deformed structures.

Fig. S11

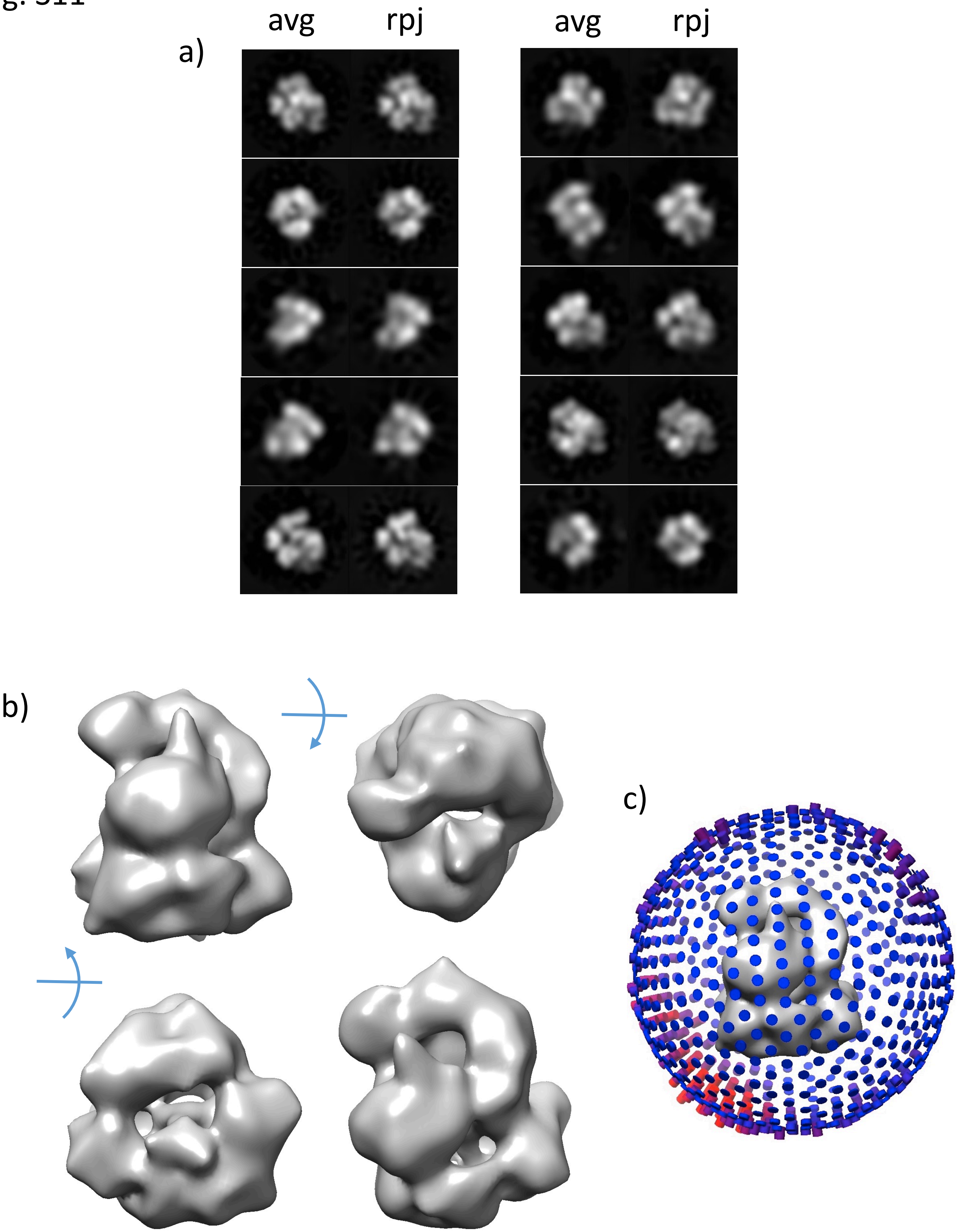

Validation of the FEN-Lig-PCNA-DNA complex structure analysis (Class 1)

a) Comparison between reference-free class averages (avg: left) and re-projections (rpj: right) of the refined map of FEN-Lig-PCNA-DNA. The height of each image box is 20.8 nm. b) The 3D map of the FEN-Lig-PCNA-DNA complex. Front view (upper left), top view (upper right), bottom view (lower left), and oblique view (lower right). c) Angular distribution plot for the refined map.

Fig. S12

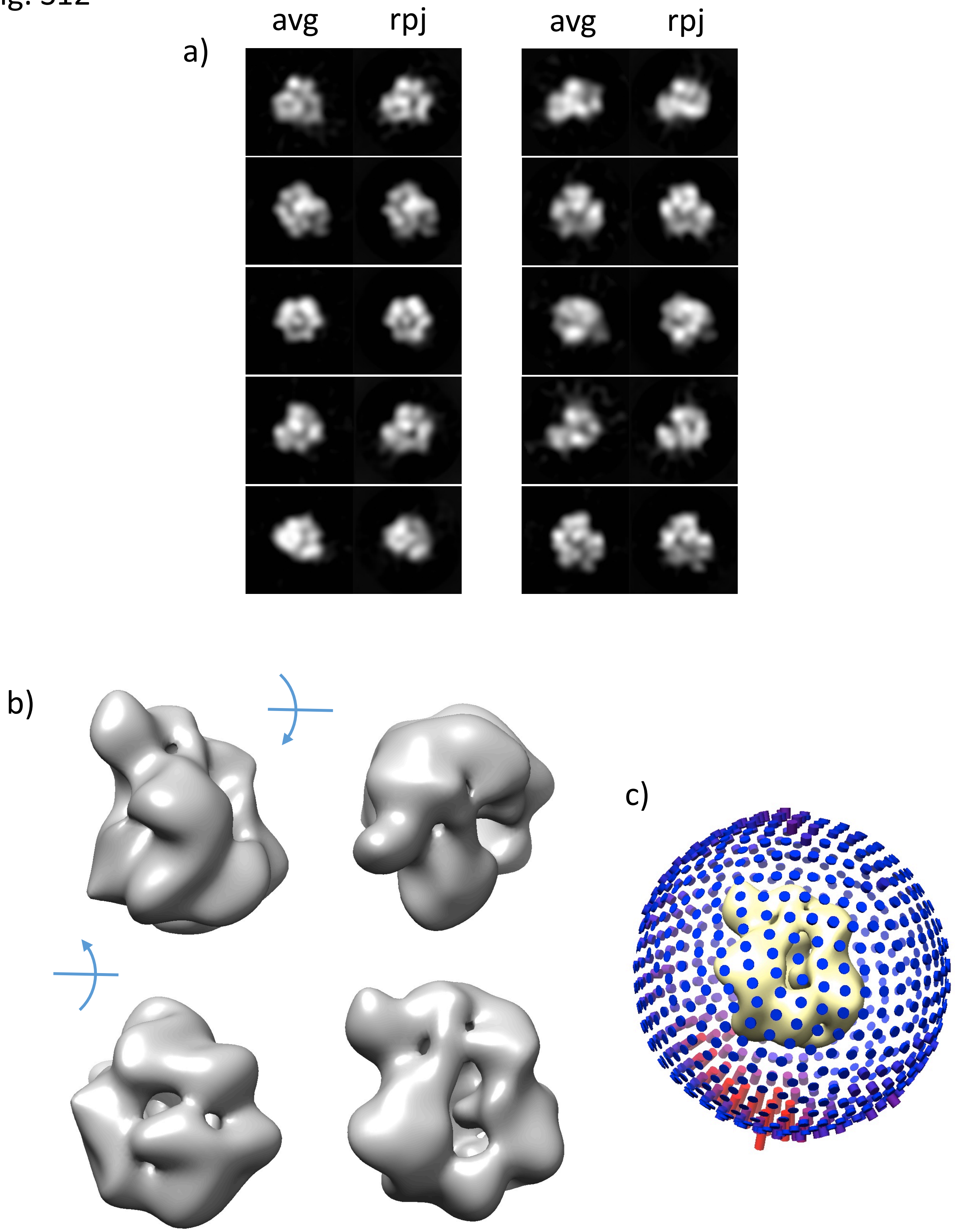

Validation of the FEN-Lig-PCNA-DNA complex structure analysis (Class 2)

a) Comparison between reference-free class averages (avg: left) and re-projections (rpj: right) of the refined map of FEN-Lig-PCNA-DNA. The height of each image box is 20.8 nm. b) The 3D map of the FEN-Lig-PCNA-DNA complex. Front view (upper left), top view (upper right), bottom view (lower left), and oblique view (lower right). c) Angular distribution plot for the refined map.

Fig. S13

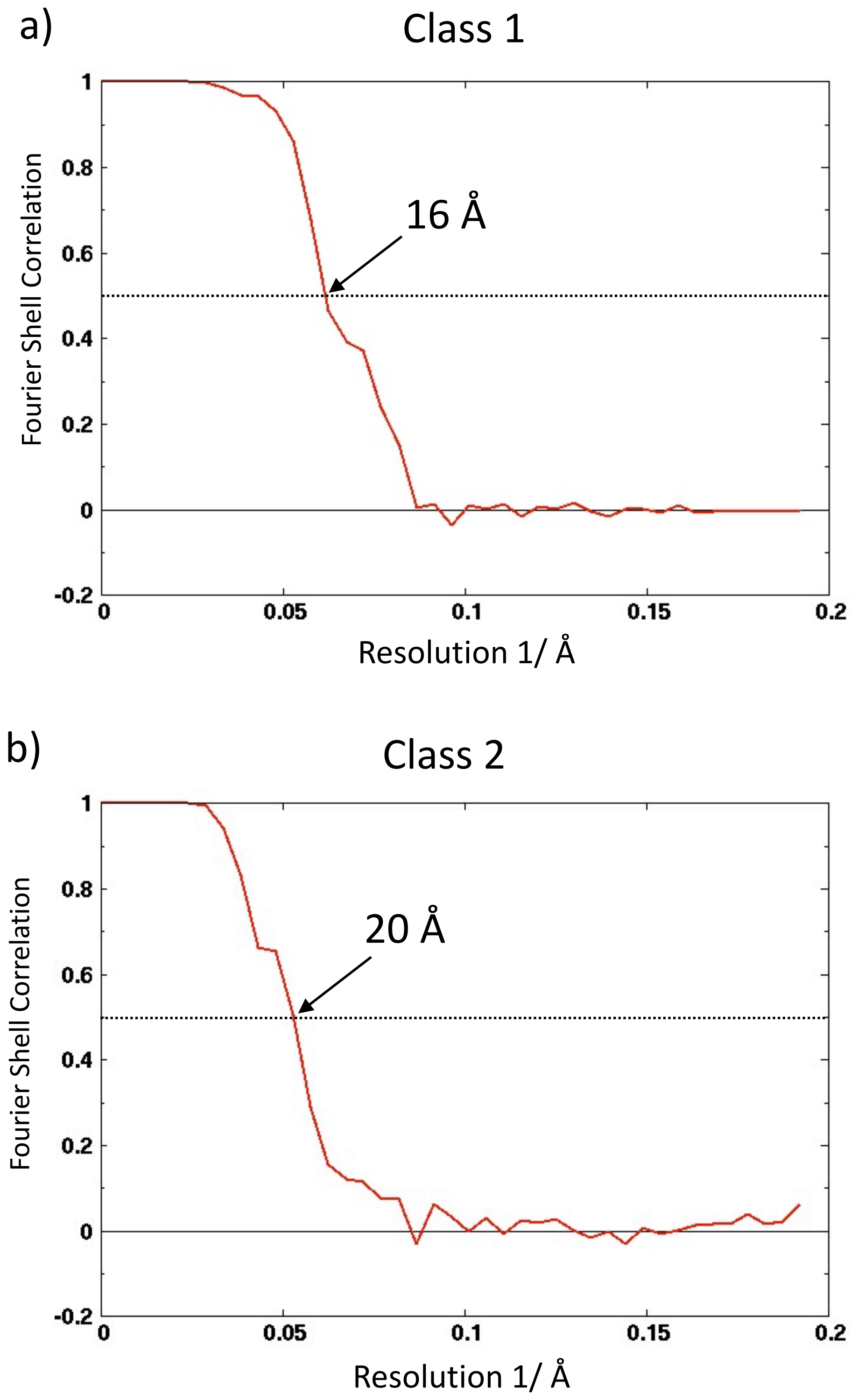

Fourier shell correlation for the FEN-PCNA-DNA complex

a) Fourier shell correlation (FSC) for the FEN-Lig-PCNA-DNA complex (class 1). The correlation between two independently refined halves of the data is indicated by the solid red line, (gold-standard FSC). The resolution estimated at a correlation of 0.5 is 16 Å. b) FSC for class 2 complex. The estimated resolution is 20 Å (FSC = 0.5).

Fig. S14

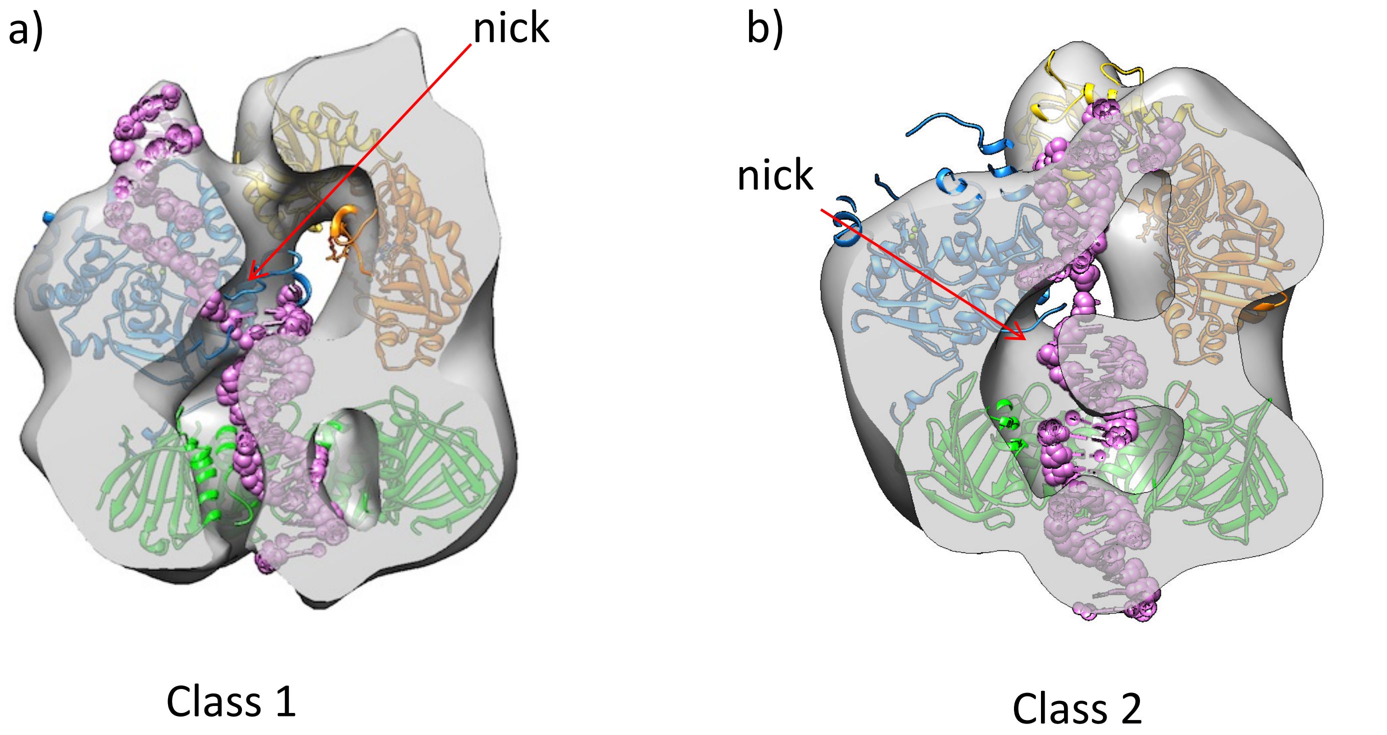

Cross sections of the 3D maps of the FEN-Lig-PCNA-DNA complex

a) Cross section of class 1. The upstream DNA (below the nick) is visualized successfully as a continuous rod. b) Cross section of class 2. The DNA density in the PCNA channel becomes weak in the middle.

Fig. S15

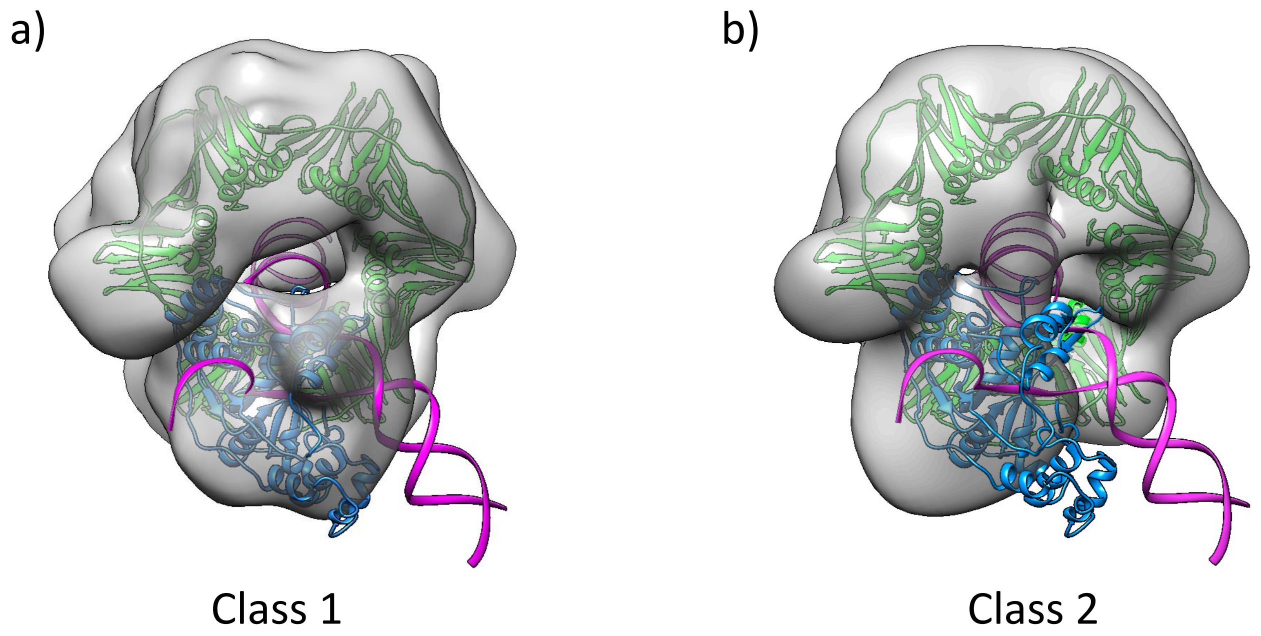

Comparison of the FEN-Lig-PCNA-DNA complex with the FEN-PCNA-DNA atomic model
The atomic model of the FEN-PCNA-DNA complex (Fig. 3) was fitted to the two classes of the FEN-Lig-PCNA-DNA complex. a) Class 1. b) Class 2. The atomic models of PCNA, FEN, and DNA are shown by green, blue, and magenta ribbon models, respectively.

Fig. S16

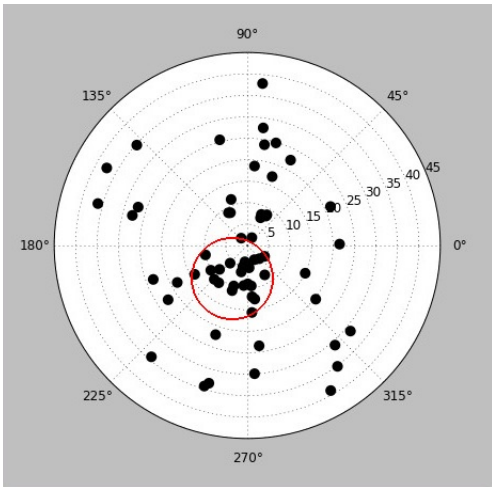

Tilt validation plots of of the FEN-Lig-PCNA-DNA structure (class 2).
Tilt validation analysis of FEN-Lig-PCNA-DNA structure, using 10˚ tilted – untilted image pairs and class 2 map of the complex. A cluster of plots (red circle) were found at the α = 8.5˚ tilt angle, close to the expected α = 10˚ nominal tilt angle.
